# Annotating Protein Functional Residues by Coupling High-Throughput Fitness Profile and Homologous-Structure Analysis

**DOI:** 10.1128/mBio.01801-16

**Published:** 2016-11-01

**Authors:** Yushen Du, Nicholas C. Wu, Lin Jiang, Tianhao Zhang, Danyang Gong, Sara Shu, Ting-Ting Wu, Ren Sun

**Affiliations:** a Department of Molecular and Medical Pharmacology, University of California Los Angeles, Los Angeles, California, USA; b Cancer Institute, Collaborative Innovation Center for Diagnosis and Treatment of Infectious Diseases, ZJU-UCLA Joint Center for Medical Education and Research, Zhejiang University School of Medicine, Zhejiang University, Hangzhou, Zhejiang, China; c Molecular Biology Institute, University of California Los Angeles, Los Angeles, California, USA; d Department of Neurology, University of California Los Angeles, Los Angeles, California, USA

## Abstract

Identification and annotation of functional residues are fundamental questions in protein sequence analysis. Sequence and structure conservation provides valuable information to tackle these questions. It is, however, limited by the incomplete sampling of sequence space in natural evolution. Moreover, proteins often have multiple functions, with overlapping sequences that present challenges to accurate annotation of the exact functions of individual residues by conservation-based methods. Using the influenza A virus PB1 protein as an example, we developed a method to systematically identify and annotate functional residues. We used saturation mutagenesis and high-throughput sequencing to measure the replication capacity of single nucleotide mutations across the entire PB1 protein. After predicting protein stability upon mutations, we identified functional PB1 residues that are essential for viral replication. To further annotate the functional residues important to the canonical or noncanonical functions of viral RNA-dependent RNA polymerase (vRdRp), we performed a homologous-structure analysis with 16 different vRdRp structures. We achieved high sensitivity in annotating the known canonical polymerase functional residues. Moreover, we identified a cluster of noncanonical functional residues located in the loop region of the PB1 β-ribbon. We further demonstrated that these residues were important for PB1 protein nuclear import through the interaction with Ran-binding protein 5. In summary, we developed a systematic and sensitive method to identify and annotate functional residues that are not restrained by sequence conservation. Importantly, this method is generally applicable to other proteins about which homologous-structure information is available.

## IMPORTANCE

To fully comprehend the diverse functions of a protein, it is essential to understand the functionality of individual residues. Current methods are highly dependent on evolutionary sequence conservation, which is usually limited by sampling size. Sequence conservation-based methods are further confounded by structural constraints and multifunctionality of proteins. Here we present a method that can systematically identify and annotate functional residues of a given protein. We used a high-throughput functional profiling platform to identify essential residues. Coupling it with homologous-structure comparison, we were able to annotate multiple functions of proteins. We demonstrated the method with the PB1 protein of influenza A virus and identified novel functional residues in addition to its canonical function as an RNA-dependent RNA polymerase. Not limited to virology, this method is generally applicable to other proteins that can be functionally selected and about which homologous-structure information is available.

## INTRODUCTION

**FIG 1:**
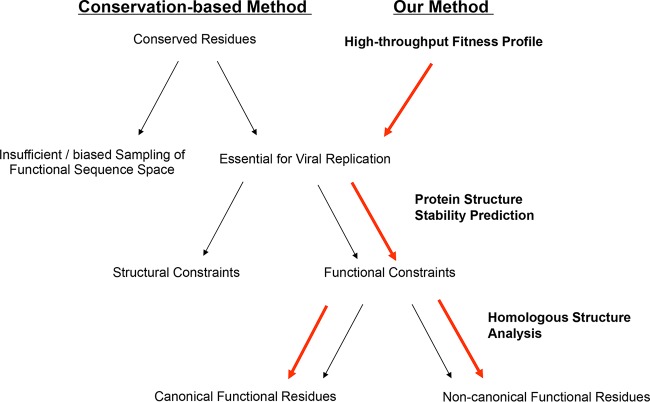
Comparison of the conservation-based method and our method. The conservation-based method is commonly used to identify and annotate functional protein residues, but it has three major limitations. First, it is limited by the insufficient sampling of protein functional space in natural evolution. Second, it is challenging for this method to dissect residues with structural or functional constraints. Lastly, it is limited to distinguishing the diverse functions within the same protein. The method we present here may overcome these limitations and provide a systematic way to annotate functional residues. Using high-throughput fitness profiling, we can identify essential residues for viral replication. Through mutant protein stability prediction, we are able to dissect the structural and functional constraints. Homologous structural analysis is used to further annotate canonical and noncanonical functional residues.

Amino acid residues in a protein have two roles: providing a structural framework (structural residues) and mediating interactions with other biomolecules (functional residues). Identification and annotation of functional residues are fundamental goals in protein characterization (1–5). A number of methods have been developed to achieve these goals. Most methods use sequence conservation information, with the assumption that functional residues are often conserved in homologous proteins (6–8). The residues identified are then expected to perform functions similar to those of other homologs. Other methods predict functional residues on the basis of the shapes and properties of three-dimensional protein structures (9–15). Starting from well-known functional domains (ligand binding, catalytic, etc.), these analyses determine similar local structures and key residues that may be related to the function. Conservation-based methods provide valuable information on protein functional residues but are limited by the insufficient sampling of protein sequence space in natural evolution. It is also challenging for conservation-based methods to assess structural and functional constraints and to assign functionality at the single-residue level (Fig. 1) (16). Therefore, a more direct and systematic method needs to be used for the accurate identification and annotation of functional residues.

Because of their compact genome, viruses usually encode multifunctional proteins, including viral polymerase proteins. Viral RNA-dependent RNA polymerase (vRdRp) is used by many RNA viruses for transcription and replication. Functions of vRdRp can be grouped into two classes: canonical and noncanonical. The canonical vRdRp functions include template and nucleotide binding, initiation, and elongation (17–19). Among different classes of RNA viruses, these canonical functions and corresponding protein structural features are conserved (17–22). The noncanonical functions of vRdRp, however, are specific to each virus. For example, multimerization of hepatitis C virus (HCV) vRdRp is essential for viral replication. Thus, the interacting residues in HCV vRdRp are noncanonical functional residues specific to HCV (23, 24). Moreover, vRdRp often recruits cellular machinery for replication and plays a role in inhibition of the cellular immune response (25–31). Noncanonical functional residues are usually involved in the performance of those functions and thus are essential for viral replication. Noncanonical functional residues in vRdRp are difficult to determine by commonly used methods and are not as well studied as the key residues for polymerase catalytic functions. However, the noncanonical functional residues are indispensable for thorough protein characterization and may act as drug targets. As a result, it is essential to develop methods that enable the identification of noncanonical functional residues.

We previously developed a method to systematically identify functional residues by coupling experimental fitness measurement with protein stability prediction (16). Here, we extended this method to annotate functional residues in combination with structural comparison of homologous proteins. The method consists of three steps. First, the effect of PB1 mutations on viral replication at single-nucleotide resolution is examined by saturation mutagenesis and high-throughput sequencing. Second, functional PB1 residues that are essential for viral growth but do not affect protein stability are identified by protein stability prediction. Third, homologous structural alignment is used to further annotate specific biological functions (canonical versus noncanonical functions) for each functional residue (Fig. 1). We achieved high sensitivity in identifying and annotating the canonical polymerase functional residues. Moreover, we also identified noncanonical functional residues, which are exemplified by a cluster of residues located in the loop region of the PB1 β-ribbon. These previously uncharacterized residues were shown to be important for PB1 protein nuclear import by interacting with Ran-binding protein 5 (RanBP5) (32).

## RESULTS

### Fitness profile of influenza A/WSN/33 virus segment 2 at single-nucleotide resolution.

**FIG 2:**
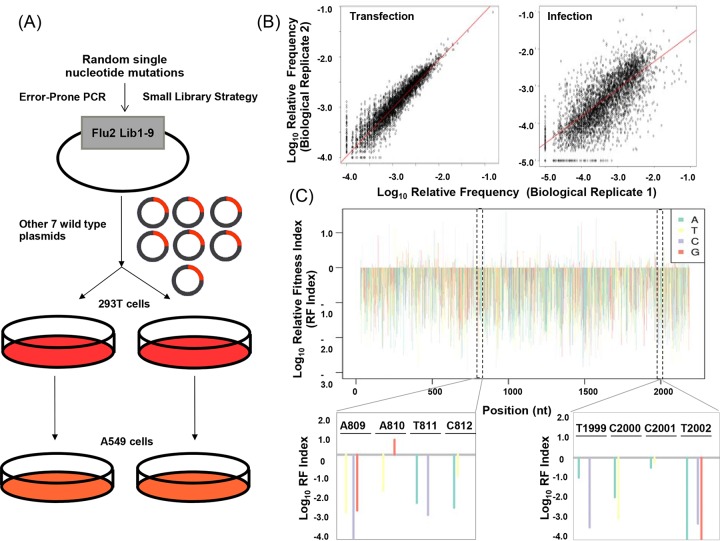
Fitness profile of influenza A virus segment 2 at single-nucleotide resolution. (A) Schematic representation of the experimental flow of high-throughput fitness profiling. Random single nucleotide mutations were introduced into influenza A/WSN/33 virus segment 2. Mutant viral libraries were generated by cotransfecting a mutant DNA library with seven plasmids encoding the other wild-type viral fragments. Viral libraries were then passaged in A549 cells. High-throughput sequencing of the plasmid mutant libraries and posttransfection and postinfection viral libraries was performed. (B) Correlation of the relative frequency of each single-nucleotide mutation between biological duplicates. (C) RF scores of individual mutations of influenza A/WSN/33 virus segment 2 in log_10_. Two representative regions are zoomed in on to show the single nucleotide change.

High-throughput genetics have been applied to a number of viral, bacterial, and cellular proteins (16, 33–38, 111, 112). Here, point mutations were randomly introduced into segment 2 of influenza A/WSN/33 virus through error-prone PCR. To provide a more accurate quantification of the fitness effect of single mutations, we employed the “small-library” method that we recently developed (16). Nine small libraries were generated to cover all of segment 2 (see Fig. S1A in the supplemental material). Each small library was transfected into 293T cells together with seven plasmids that encoded the other wild-type viral segments (39). Reconstituted mutant virus libraries were used to infect A549 cells at a multiplicity of infection (MOI) of 0.05, and supernatants were collected 24 h postinfection. The input DNA libraries, posttransfection libraries, and postinfection libraries were subjected to Illumina sequencing. To control for technical error and assess library quality, biological duplicates were included in both transfection and subsequent infection steps (Fig. 2A).

The distribution of the number of mutations in the input DNA library was examined. Thirty to 35% of the input DNA library plasmids contained the desired single nucleotide mutations (see Fig. S1B in the supplemental material). We achieved at least 20,000× sequencing coverage for each nucleotide position (see Fig. S1C). The library covered 94.9% of the nucleotides in segment 2 and included 98.2% of the single nucleotide mutations of observed positions (see Fig. S2A and C). To further improve the accuracy of fitness quantification, we focused on the mutations that make up >0.1% of the plasmid mutant library. After this quality control, we were still able to observe 94.2% of the nucleotide positions with 63.9% of the single nucleotide mutations. More than 82% of the nucleotide positions were covered with two or three nucleotide mutations (see Fig. S2B and D in the supplemental material). To assess the quality and reproducibility of our mutant library, we compared the relative frequencies of single mutations between biological replicates. We obtained a strong Spearman correlation coefficient of 0.93 for two independent transfections and 0.75 for infections (Fig. 2B). A relative fitness (RF) index was calculated for individual mutations as the ratio of relative frequency in the infection library to that in the input DNA library. The profiling data of all of segment 2 are shown in Fig. 2C, where most of the mutations had a fitness cost (log_10_ RF index of <0).

### Systematic identification of deleterious mutations of the PB1 protein.

Segment 2 of influenza A virus encoded three proteins: PB1, PB1-F2, and N40. N40 was a truncated form of the PB1 protein that lacked the first 39 amino acids. PB1-F2 is not essential for viral replication *in vitro*, as completely abolishing PB1-F2 expression had no effect on viral growth (40, 41) (see Fig. S3 in the supplemental material). So we focused on the PB1 protein for downstream analysis. The RF indexes of silent mutations were considered an internal quality control since most, if not all, of them were expected to have a growth capacity comparable to that of the wild type. In the fitness profile of the PB1 protein, the RF indexes of silent mutations followed a normal distribution with a mean of 0.9 and were significantly higher than those of nonsense mutations (two-tailed *t* test, *P* = 4.6E-21) (see Fig. S4 in the supplemental material). This result confirms the presence of fitness selection and validates the data quality.

**FIG 3:**
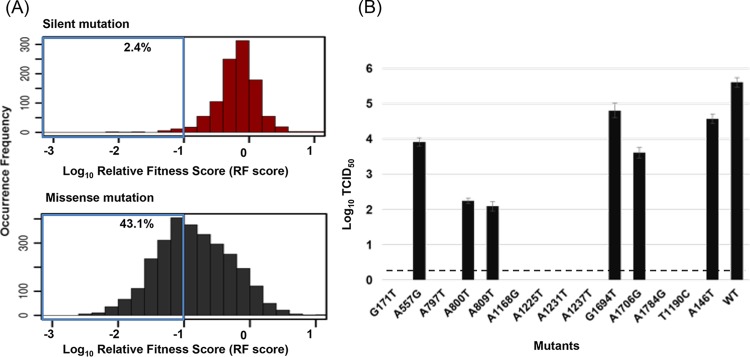
Systematic identification of deleterious mutations of the PB1 protein. (A) Histogram illustrations of the RF distribution (RF index in log_10_) of silent and missense mutations. Mutations with an RF index of ≤0.1 were identified as deleterious mutations. The percentages of silent and missense mutations that fall below this cutoff are boxed in blue. (B) Fourteen deleterious mutations were selected and reconstructed in the viral genome. The TCID_50_s of selected single nucleotide mutations are shown. The dashed line represents the detection limit of the TCID_50_ assay. Data are presented as mean values ± standard deviations of biological duplicates. WT, wild type.

To systematically identify deleterious mutations, we chose a stringent RF index cutoff of ≤0.1. A total of 2.4 percentage points of silent mutations fell below the cutoff, which represented type I error. A total of 43.1 percentage points of missense mutations that satisfied this cutoff were identified as deleterious mutations (Fig. 3A). We randomly selected 14 deleterious mutations and reconstructed them individually. Rescue experiments were performed, and the resultant viral titers were quantified by 50% tissue culture infective dose (TCID_50_) assay. Thirteen of 14 mutant viruses had at least a 10-fold drop in the viral titer compared to that of the wild type. The other mutant also showed a more-than-6-fold titer decrease (Fig. 3B). These results validated the approach we used to systematically quantify the RF and identify deleterious mutations of the PB1 protein.

### Identifying functional residues by dissecting structural and functional constraints.

A mutation might be deleterious because of structural or functional constraints (16, 42). We have recently demonstrated that coupling high-throughput genetics with mutant stability predictions can identify residues that are dominated by functional constraints (16). Briefly, deleterious mutations that do not destabilize the protein are identified as functional residues. Here, we modeled protein stability by using two computational tools: I-Mutant and Rosetta ddg monomer (see Data Set S1 in the supplemental material).

I-Mutant is a supporter vector machine-based software used to predict the effect of single-site mutations on protein stability (ΔΔG) (43–45). On the basis of the predicted ΔΔG, mutations can be classified as destabilizing (ΔΔG, ≤−0.5), neutral (−0.5 < ΔΔG < 0.5), or stabilizing (ΔΔG, ≥0.5). We applied I-Mutant predictions for all missense mutations in PB1 with the structure resolved from the bat influenza A virus polymerase complex (Protein Data Bank [PDB] code 4WSB) (46, 47). Of the mutations for which structure information is available, 64.5% were shown to be destabilizing, 33.5% were neutral, and 2% were stabilizing (see Fig. S5A in the supplemental material). As expected, destabilizing mutations had a significantly small solvent-accessible surface area (SASA) (48–50) (see Fig. S5B). To further reduce the rate of false-negative functional residue identification, we performed protein stability prediction with Rosetta for all deleterious mutations (16, 42, 44). Unlike the machine learning algorithm used by I-mutant, Rosetta generated structural models for single amino acid mutations based on a preoptimized wild-type structure. With a high-resolution protocol, 50 models of wild-type and mutant protein structures were generated and the three lowest ΔΔG values were averaged on the basis of optimized rotamers. The absolute correlation coefficient of the predictions that resulted from these two methods was 0.3 (see Fig. S5C). Aiming at getting a conserved classification of functional residues, we classified a residue as functional if it had one or more missense mutations satisfying both the deleterious RF index cutoff and nondestabilizing criteria of ΔΔG predictions from either software. We identified 297 residues as functional.

To examine the sensitivity of our method of identifying functional residues in PB1, we performed a thorough literature search, compiled 31 residues that were reported to be functional in PB1 (32, 51–54), and compared the performance of our method with that of four other methods: FireStar, Frpreq, Consurf, and Concavity (6, 10, 55–58) (Table 1). Our method was able to identify 21 of the 31 residues and thus had a sensitivity of ~68%. FireStar failed to identify any of them. Frprep, Concavity, and Consurf identified 4 (Frprep score, ≥8), 7 (Concavity score, >0.1), and 17 (Consurf score, 9) residues, respectively. Notably, our method was the only one that identified functional residues related to noncanonical polymerase functions (four of the eight residues) that were not conserved in sequence or structure. Overall, these results validated our method of combining high-throughput genetics with mutant stability prediction to identify functional residues in PB1 in a sensitive and unbiased manner (16, 42, 44).

**TABLE 1:** Comparison of methods of identification of known functional PB1 residues

Mutation	Functional annotation	Our method	FireStar	Frpred	Consurf	ConCavity
L8	Interact with PA	0	0	1	3	0
F9	Interact with PA	0	0	1	3	1.40E-6
L10	Interact with PA	0	0	1	6	0
K11	Interact with PA	1	0	1	5	0
M179	Polymerase activity	0	0	2	4	4.40E-8
K188	Nuclear localization	1	0	2	6	0
R189	Nuclear localization	1	0	1	3	0
R208	Nuclear localization	1	0	1	1	0
K209	Nuclear localization	0	0	2	3	0
K229	Polymerase activity	1	0	7	9	0.288
R233	Polymerase activity	0	0	7	9	0.044
K235	Polymerase activity	1	0	7	9	0.682
R238	Polymerase activity	1	0	7	9	0.201
R239	Polymerase activity	0	0	7	9	0.187
K278	Polymerase activity	1	0	6	9	0.022
K279	Polymerase activity	1	0	6	9	1.08E-5
N306	Polymerase activity	1	0	6	8	0.437
K308	Polymerase activity	1	0	6	9	0.027
M409	Polymerase activity	1	0	9	9	0.829
Q442	Polymerase activity	1	0	4	9	0.653
S444	Polymerase activity	1	0	7	9	0.009
D445	Polymerase activity	1	0	6	9	0.001
D446	Polymerase activity	1	0	8	9	5.25E-6
N476	Polymerase activity	1	0	7	9	0.008
S478	Polymerase activity	0	0	7	9	0.011
K481	Polymerase activity	1	0	8	9	0
Y483	Polymerase activity	1	0	4	8	0
E491	Polymerase activity	1	0	8	9	0.028
F492	Polymerase activity	1	0	6	8	0.001
F496	Polymerase activity	0	0	5	8	0.001

### Annotating functional residues by homologous structural alignment.

The vRdRp family has a conserved “right-handed” structure. It consists of three major conserved domains (finger, palm, and thumb) and six motifs (pre-A/F and A to E) (20). Since canonical vRdRp functional residues of the PB1 protein are expected to be structurally conserved, they aligned well with other protein structures from the vRdRp family. Therefore, homologous structural alignment might enable us to further annotate PB1 residues by distinguishing canonical and noncanonical vRdRp functional residues. The recent improvement of algorithms provides opportunities for more accurate structure comparison. Here we used TM-align and 3DCOMB for pairwise and multiple structure alignments (MSAs) (59–61). Both softwares use TM-score to quantify protein structural similarity, which is robust to local structural variation and is protein length independent (59, 60). Moreover, 3DCOMB takes into account both local and global features, which is suitable for alignment of distantly related protein structures (61).

Twenty representative vRdRp structures were selected from positive single-stranded RNA (ssRNA) viruses, negative ssRNA viruses, and double-stranded RNA (dsRNA) virus families on the basis of previously stated criteria (20). Briefly, representative structures were selected from each of the Baltimore classes that encoded vRdRp, including positive ssRNA viruses (*Caliciviridae*, *Flaviviridae*, *Picornaviridae*, *Cystoviridae*), dsRNA viruses (*Birnaviridae*, *Cystoviridae*, and *Reoviridae*), and negative ssRNA viruses (*Bunyaviridae*) (62–81). Structures with no mutations and with a bound substrate were preferred. PDB files with the highest resolution were picked for each protein (see Table S1 in the supplemental material).

To ensure sufficient structural similarity, a pairwise structural comparison was performed with the selected protein and PB1 by using TM-align. The structures with TM scores of >0.5 were kept for multiple structural alignment, which generally indicated similar protein folding (43). Figure S6A in the supplemental material provides an example superimposition of the PB1 protein with HCV NS5B (PDB code 2XI3) with decent alignment in major protein domains (67). A total of 16 proteins were included for MSA with PB1 by using 3DCOMB (see Table S1 in the supplemental material).

**FIG 4:**
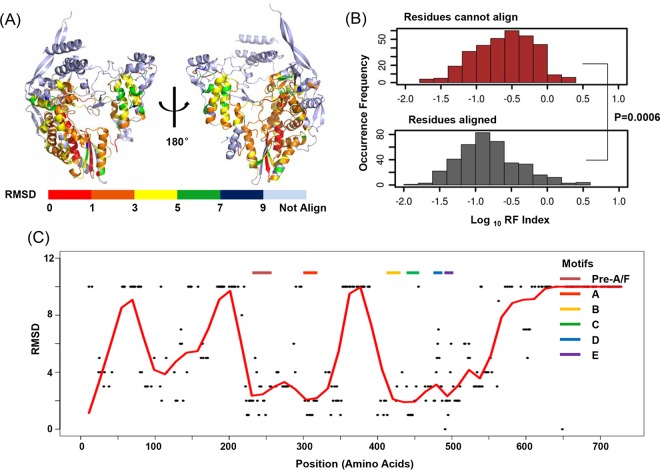
Annotation of PB1 functional residues with homologous structural alignment. (A) An MSA was performed with PB1 and 16 other homologous structures in the vRdRp family. The PB1 structure is rainbow colored according to the RMSD of each residue. (B) Histograms of the RF indexes are shown for residues that cannot be aligned (red) and residues that can be aligned with other structures in the vRdRp family. The RF indexes of residues that cannot be aligned were significantly higher (two-tailed *t* test, *P* = 0.0006). (C) RMSDs of functional residues. A smooth curve was fitted by loess regression. Conserved vRdRp domains (pre-A/F and A to E) are labeled and shown as valleys on the smooth RMSD curve.

The root mean square deviation (RMSD), the measurement of the average distance between the atoms and superimposed proteins, was reported by 3DCOMB for each residue as the representative of structure conservation. As the reported aligned residues had RMSD scores ceiled at 9, we assigned the residues that did not align among structures with an RMSD value of 10 (Fig. 4A). Low RMSDs meant that the residues were conserved in the vRdRp family and thus more likely to have canonical vRdRp functions. As expected, the structurally conserved residues were less tolerant of mutations. The average RF index of structurally conserved residues was significantly lower than that of nonconserved residues (two-tailed *t* test, *P* = 0.0006, Fig. 4B). The RMSDs of all of the identified functional residues of the PB1 protein were plotted. A smooth curve of RMSDs was fitted by local polynomial (loess) regression. We could clearly identify the six conserved domains (pre-A/F and A to E) of vRdRp as valleys on the smooth curve (Fig. 4C). These results demonstrated the feasibility of using homologous structural alignment to identify canonical vRdRp residues.

### Identification of noncanonical functional residues, ones involved in nuclear import of the PB1 protein.

Forty-three percent of the functional residues identified could not be aligned with other protein structures from the vRdRp family. Although this could be due to poor alignment quality, it is also possible that these residues have noncanonical functions that are essential for viral growth. Interestingly, 62% of these residues belong to the protein interface between PB1 and PB2 or PA, as identified by the change in SASA upon complex formation by using Sppider (residues with at least a 4% decrease in SASA and >5 Å^2^ of exposed surface area upon complex formation) (82) (see Fig. S6B in the supplemental material). These interface residues also accounted for some of the peaks (residue 50 to 80, residues 350 to 400, and residues at the C terminus of PB1) in the smooth RMSD curve of functional residues in Fig. 3C.

**FIG 5:**
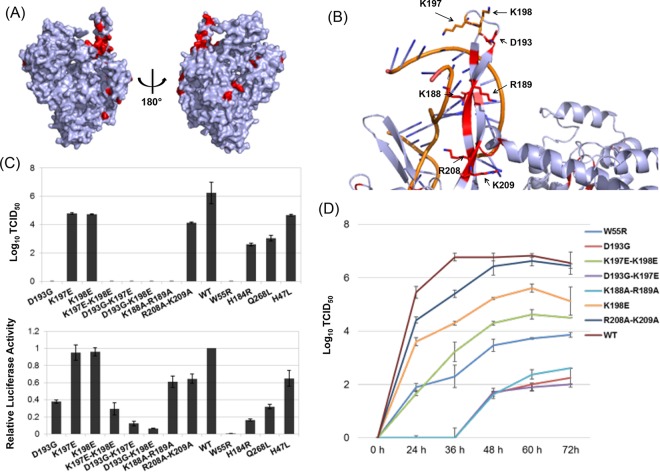
Identification of noncanonical functional residues of the PB1 protein. (A and B) Noncanonical noninterface functional residues of the PB1 protein are red. A cluster of residues is located in the long twisted β-ribbon region. The nonstructured loop region (amino acids 195 to 198) was reconstructed with Rosetta. (C) TCID_50_s (top) and relative polymerase activities (bottom) of the mutations indicated. The data are presented as mean values ± standard deviations of four independent biological replicates. (D) Growth curves of the mutations indicated. A549 cells were infected with the mutant viruses indicated at an MOI of 0.1. Viruses were collected at the time points indicated, and TCID_50_s were measured. WT, wild type.

We then performed a detailed analysis of the noncanonical functional residues that were not located in the heterotrimer-forming interface. When mapped onto the protein structure, some of them (residues 180 to 220) formed a noticeable cluster (Fig. 5A and B). This clustered region is unique to the PB1 protein, which consists of a long twisted β-ribbon connected by a nonstructured loop (47). It protrudes from the polymerase complex structure and is fully solvent exposed. Two nuclear localization signals (NLSs) were reported in the β-ribbon region (amino acids 187 to 190 and 207 to 210) to mediate PB1 nuclear import through interaction with RanBP5 (32, 83). Nonetheless, the function of this loop region is not completely clear. It is suspected to interact with the viral genome in the resolved influenza B and C virus structures (46, 47, 84), and K198 of influenza A virus was suggested to be related to host adaptation (85). As the density of the loop region (residues 195 to 198) is missing from the influenza A virus polymerase crystal structure, we used kinematic loop modeling in the Rosetta software to computationally reconstruct the loop region (86). From the above-described analysis, D193 in the loop region was identified as a noncanonical functional residue. Interestingly, it was the only negatively charged residue located within a highly positively charged environment. It was 100% conserved among all of the human influenza A virus PB1 sequences from the Influenza Research Database under purifying selection (ratio of nonsynonymous to synonymous evolutionary changes [*dN*/*dS* ratio] of 0.015) (87–89, 113) (see Table S2 in the supplemental material). Two positively charged residues (K197, K198) located on the side opposite D193 in the loop region were also highly conserved in human influenza A viruses (>99%) and possibly interact with D193. Although they were not classified as essential residues according to our high-throughput fitness profile, their mutations in charges (K197E, K198E) resulted in a >6-fold drop in the RF index. To examine if the loop region has possible noncanonical functions, we introduced single substitutions (D193G, K197E, K198E) and double substitutions (D193G-K197E, G193G-K198E, K197E-K198E) into the PB1 protein. We also constructed mutant versions with substitutions in the NLS region (K188A-R189A, R208A-K209A) and mutant versions that decreased the polymerase activity (W55R, H184R, H47L, Q268L) as controls. Of note, all of the controls were identified as deleterious in our high-throughput fitness profile. Viral production of all of the mutant versions was measured by TCID_50_ assay with viral rescue experiments. D193G, D193G-K197E, G193G-K198E, K197E-K198E, and the reported substitutions in the NLS region (K188A-R189A) had severe impacts on viral production, with no detectable viral titer posttransfection (Fig. 5C) (32). Consistently, these mutations also resulted in a significantly lower viral growth rate in A549 cells (Fig. 5D). To examine the vRdRp function of these mutations, we used a minigenome replicon assay by cotransfecting a virus-inducible luciferase reporter and polymerase segments (PB2, PB1, PA, NP) in 293T cells. The reported mutant NLS (K188A-R189A), which was highly deleterious for viral replication, still had ~50% polymerase activity in the minigenome replicon assay. Similarly, D193G and all of the double substitutions (D193G-K197E, D193G-K198E, K197E-K198E) showed discordance between vRdRp function and viral growth capacity. Compared with W55R, H184R, H47L, and Q268L, which remained at ~0.1 to 65% polymerase activity, the fitness drop caused by these newly identified loop mutations was much more severe, indicating that they might have a noncanonical polymerase function of PB1 (Fig. 5C).

**FIG 6:**
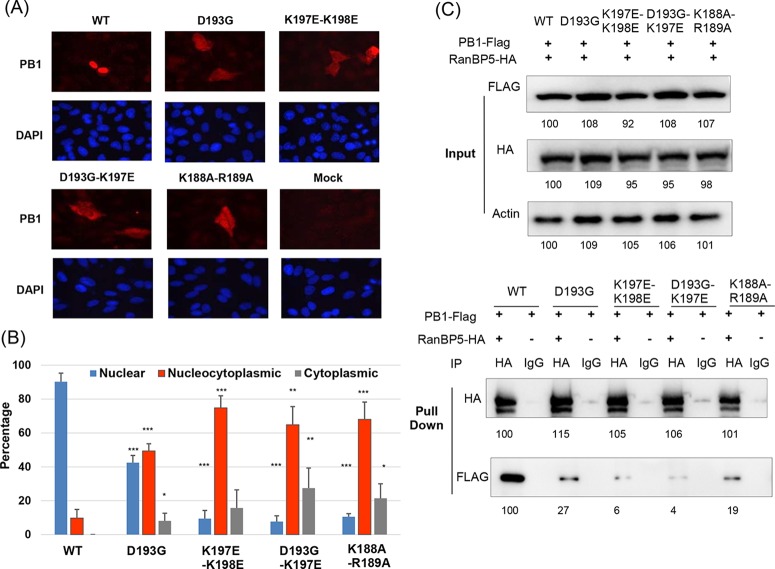
The noncanonical functional residues identified may be involved in nuclear import of the PB1 protein by interaction with RanBP5. (A) Cellular localizations of wild-type (WT) and mutant PB1 proteins determined by IFA. (B) Percentages of cells with different PB1 localizations. Data are presented as mean values ± standard deviations of three independent biological replicates. At least 50 cells of each replicate were analyzed with ImageJ. *, *P* < 0.05; **, *P* < 0.01; ***, *P* < 0.001 (two-tailed *t* test). (C) Interactions between PB1 proteins and RanBP5 were examined by IP. The value below each band is the intensity quantification measured by Image Lab.

Unlike other RNA viruses, the genome replication and transcription of influenza virus are performed inside the nucleus. Nuclear localization function is thus specific to influenza virus and belongs to noncanonical functions of the PB1 protein. We tested if the mutations identified in the loop region (D193G, D193G-K197E, K197E-K198E) had effects on protein nuclear import. A549 cells were infected with wild-type and mutant viruses at an MOI of 0.1. Cells were fixed and subjected to immunofluorescence analysis (IFA) at 18 h postinfection. As expected, the PB1 proteins of the wild-type virus were localized mostly in the nucleus. However, the PB1 proteins of mutant viruses were significantly enriched in the cytoplasm, suggesting that these mutations were defective in PB1 protein nuclear import (Fig. 6A and B). More severe defects were observed for double mutations (D193G-K197E, K197E-K198E). Similar results were observed at earlier time points (8 h postinfection) at an MOI of 0.5 (see Fig. S7 in the supplemental material). Interestingly, for those PB1 mutant versions, the nuclear import of PA protein was also delayed, which is consistent with the notion that PA and PB1 are imported into the nucleus as a complex (32, 83, 90, 91) (see Fig. S7).

RanBP5 belongs to the importin-β family, which has a nonclassical nuclear import function (92, 93). RanBP5 has been shown to be important for influenza A virus PB1 nuclear import. The NLS mutations affected protein nuclear import by decreasing binding to RanBP5 (32, 83, 92). Thus, we further tested if mutations in the loop region (D193G, D193G-K197E, and K197E-K198E) would also affect the interaction between PB1 and RanBP5. Immunoprecipitation (IP) was performed by cotransfecting the FLAG-tagged PB1 protein and the hemagglutinin (HA)-tagged RanBP5 protein into 293T cells. Two days later, the total cell lysate was collected and subjected to IP with anti-HA antibody-conjugated beads or IgG-conjugated beads. As shown in Fig. 6B, all three mutant proteins showed decreased binding with RanBP5. Consistent with our IFA results, double mutations (D193G-K197E, K197E-K198E) produced a greater reduction in protein binding. The above-described results indicate that the residues in the loop region are important for nuclear import of the influenza A virus PB1 protein through interaction with RanBP5, which is a noncanonical function in the vRdRp family.

## DISCUSSION

For a comprehensive characterization of protein function, identification and annotation of functional residues are the fundamental tasks. Here we present a systematic approach to these tasks by using influenza A virus PB1 as the target protein. Our approach combines high-throughput fitness profiling with mutant stability prediction and homologous structural alignment to identify and annotate canonical and noncanonical vRdRp functional residues (Fig. 1). Interestingly, we identified a cluster of mutations that were highly deleterious for viral replication but resulted in relatively intact vRdRp function. These mutations were located in the loop region of the PB1 β-ribbon and were shown to be important for PB1 nuclear import. The combination of high-throughput fitness profiling and structural analysis provided a general approach to the identification and annotation of functional residues that can be applied to a wide range of proteins about which homologous structural information is available.

In the context of evolutionary biology, proteins from the same homolog family have an ancestor in common and possess significant sequence and structural similarities (94–97). Structural similarities are postulated to be maintained by functional constraints (98, 99). vRdRps probably evolved from a common ancestor (100). Although their sequence identity is ~20%, they have adopted similar structural domains and use similar catalytic mechanisms (20). Throughout evolution, different proteins also evolved diverse functions to satisfy the needs of specific organisms. Thus, the specific structural motifs that differentiate one protein from homologous proteins may have organism-specific functions. Here we used homologous protein structure information to further annotate the diverse protein functions. Therefore, a multifunctional protein might harbor both canonical (evolutionarily conserved) and noncanonical (organism-specific) functions. The combination of high-throughput genetic screening with homologous-structure analysis enabled us to systematically understand functional residues and important single nucleotide polymorphisms.

Here we show that the residues in the loop region of the PB1 β-ribbon are important for PB1 nuclear import. Unlike other RNA viruses, influenza A virus performs its genome replication inside the nucleus. Thus, the polymerase complex needs to be translocated into the nucleus to perform its function. It is known that PB1 and PA are translocated together as a complex, while PB2 can be translocated by itself (101). RanBP5 is important for the nuclear import of PB1 and PA through direct interaction with PB1. Besides the two reported NLSs, we show that the mutations in the loop region also impact the interaction between PB1 and RanBP5, thus causing the defect in PB1 nuclear import. We do not have direct evidence that the loop region works as a direct NLS or by affecting the nearby NLS regions, but on the basis of the sequence of the loop region, it did not fall into any of the six classes of NLSs (32, 102). Thus, we suspected that this region affected PB1 nuclear import by affecting the nearby NLS regions. In agreement with previous observations, there seems to be no clear consensus sequence that is responsible or important for RanBP5 binding (32, 103). The detailed mechanism needs to be further defined.

Genetic studies are greatly facilitated by the improvement of sequencing capacity and the growing number of protein structures being resolved. Large amounts of information generated with current technologies demand more effective approaches to determine structure-function relationships. Coupling mutagenesis with high-throughput sequencing, high-throughput fitness profiling provides a sensitive and unbiased way to identify the essential residues of targeted proteins (16, 33–37, 104–107). The same principle applies to other proteins/organisms, as long as the proper functional measurement can be made (37). For example, we can study the proteins related to cell proliferation by using the cell growth rate as a readout. By using saturated mutagenesis, we can learn which mutation is related to an abnormal cell growth rate and can further use flow cytometry to differentiate cells in different phases. We can also investigate the roles of mutant proteins in cancer metastasis through transwell migration assays *in vitro* or by using mouse xenograft models *in vivo*. The structures of target or homologous proteins can be linked to a genetic profile and further facilitate the understanding of biomolecular functions related to each functional residue. We foresee that this approach will become more powerful as more protein structures are determined at an accelerated rate by crystallography and cryoelectron microscopy and the escalating sequencing technology.

In summary, we have developed a systematic and sensitive method to identify and annotate functional residues. More importantly, the method presented here is generally applicable to other proteins with structural information of homologous proteins.

## MATERIALS AND METHODS

### Construction of influenza A virus segment 2 mutant libraries.

Influenza A/WSN/33 virus segment 2 mutant libraries were generated with the eight-plasmid transfection system (39). In brief, the entire influenza virus gene was separated into nine small 240-bp segments. Random mutagenesis was performed with error-prone polymerase Mutazyme II (Stratagene). For each small library, mutagenesis was performed separately and the amplified segment was gel purified, BsaI digested, ligated to the vector, and transformed with MegaX DH10B T1R cells (Life Technologies). As each small library was expected to have ~1,000 single mutations, ~50,000 bacterial colonies were collected to cover the entirety. Plasmids from collected bacteria were midiprepped as the input DNA library.

### Transfection, infection, and viral titer.

To generate the mutant viral library, ~30 million 293T cells were transfected with 32 µg of DNA. Transfections were performed with Lipofectamine 2000 (Life Technologies). Virus was collected at 72 h posttransfection. TCID_50_s were measured with A549 cells. To passage viral libraries, ~10 million A549 cells were infected at an MOI of 0.05. Cells were washed with phosphate-buffered saline (PBS) three times at 2 h postinfection. Virus was collected 24 h postinfection from supernatant.

Individual mutant viral plasmids were generated with a quick-change system. To generate mutant virus, ~2 million 293T cells were transfected with 10 µg of DNA. To measure the growth curve, ~1 million A549 cells were infected at an MOI of 0.1 and supernatants were collected at the times indicated.

### Sequencing library construction and data analysis.

Viral RNA was extracted with the QIAamp Viral RNA Minikit (Qiagen Sciences). DNase I (Life Technologies) treatment was performed, followed by reverse transcription with the SuperScript III system (Life Technologies). At least 10^6^ viral copies were used to amplify the mutated segment. The amplified segment was then digested with BpuEI and ligated with the sequencing adaptor, which had three nucleotides multiplexing ID to distinguish between different samples.

Deep sequencing was performed with Illumina sequencing MiSeq PE250. Raw sequencing reads were demultiplexed by using the three-nucleotide ID. Sequencing error was corrected by filtering unmatched forward and reverse reads. Mutations were called by comparing sequencing reads with the wild-type sequence. Clones containing two or more mutations were discarded. The RF index was calculated for individual point mutations, and only mutations that had a frequency of >0.1% in the DNA library were reported. The formula used was *RF index_mutant i_* = *Relative Frequency of Mutant i_infection_*/*Relative Frequency of Mutant i_plasmid_*, where *Relative Frequency of Mutant i* = *Reads of Mutant i*/*Reads of wild type*.

All data processing and analysis was performed with customized python scripts, which are available upon request.

### Protein structural analysis.

Chain B (PB1 protein) of PDB code 4WSB was used for protein ΔΔG prediction with single amino acid mutations (46, 47). ΔΔG predictions were performed with both the I-Mutant 2.0 package and ddg_monomer in the Rosetta software (43, 108). Default parameters (temperature of 25°C, pH 7.0) were used in the I-Mutant package. The parameters used for Rosetta were the same as those previous described (16, 109). A ΔΔG of <0 in I-Mutant and a ΔΔG of >0 in Rosetta mean destabilization.

The DSSP tool was used to calculated SASA, which was then normalized to the empirical scale as previously described (48–50). Sppider was used to identify the protein-protein interface. Residues with at least a 4% reduction and a >5-Å^2^ reduction in SASA upon complex formation were identified as protein-protein interface residues (82).

TM-align and 3DCOMB were used for pairwise structural alignment and multiple structural alignment (59, 61). TM-score normalized to the PB1 protein was used.

### Protein loop modeling.

In the loop region of the PB1 β-ribbon, electron density for residues 195 to 198 is missing from the X-ray crystal structure (PDB code 4WSB). Rosetta software was used to computationally reconstruct the loop region, which was based on Monte Carlo sampling with exact kinematic loop closure (86). After energy optimization, each model was ranked by Rosetta full atom energy function (80). The lowest-energy model with a hairpin-like loop was selected.

### Polymerase activity assay.

One hundred nanograms each of PB2, PB1 (wild type and indicated mutations), PA, and NP; 50 ng of a virus-inducible luciferase reporter; and 5 ng of PGK-*Renilla* luciferase were transfected into 293T cells in 24-well plates (110). Cells were lysed at 24 h posttransfection, and luciferase assay was measured with the Dual-Luciferase Assay kit (Promega).

### IFA.

The localizations of wild-type PB1 and mutant PB1 proteins were determined by Immunofluorescence analysis (IFA). Infected A549 cells were fixed in 2% paraformaldehyde, permeabilized with 0.1% Triton X-100, and then blocked with 3% bovine serum albumin and 10% fetal bovine serum. Viral PB1 protein was detected with anti-PB1 antibody (GeneTex GTX125923). Hoechst 33342 dye was used for nucleic acid staining.

### IP.

Immunoprecipitation (IP) experiments were performed with HA- and FLAG-tagged proteins expressed in 293T cells. Briefly, cells were transfected with corresponding expression plasmids with Lipofectamine 2000 reagents (Invitrogen) and lysed at 2 days posttransfection with radioimmunoprecipitation assay (RIPA) buffer (50 mM Tris-HCl [pH 7.4], 0.5% NP-40, 150 mM KCl, 1 mM EDTA, protease inhibitor). Cell lysates were incubated with 1 µg of anti-HA antibody for 4 h at 4°C with constant agitation, washed with RIPA buffer five times, and eluted with 60 µl of SDS-PAGE sample buffer. All samples were subjected to SDS-PAGE and Western blotting.

### Western blotting.

Proteins in SDS-PAGE sample buffer were heated at 95°C, resolved by SDS-PAGE, and then transferred onto polyvinylidene difluoride membrane. Proteins were detected with antibodies against FLAG-epitope, HA-epitope, or actin.

### Phylogenetic analysis.

PB1 coding sequences were downloaded from the Influenza Research Database (87). Multiple sequence alignment was performed with MUSCLE (88). We randomly sampled 3,000 sequences for *dN*/*dS* calculation by Fubar with HyPhy (89).

### Accession number(s).

Raw sequencing data have been submitted to the NIH Short Read Archive under accession number PRJNA318707.

## SUPPLEMENTAL MATERIAL

Figure S1  Construction of small libraries and sequencing coverage. (A) Schematic presentation of the mutagenesis library of influenza virus segment 2, covered by nine small libraries of 240 bp each. The starting nucleotide position of each segment is labeled. (B) Distribution of mutations in the input DNA plasmid library shown as a bar chart. From ~30 to 35% of the clones in the plasmid mutant library contained one mutation (single). From ~25 to 30% were wild type, and the rest of them contained two or more mutations. (C) Sequencing depth of each small library. Download Figure S1, TIF file, 22.9 MB

Figure S2  Positions and mutation coverages of libraries. (A) Percentages of nucleotide positions that were observed in the fitness profile and percentages of total single nucleotide mutations that were observed in the fitness profile prior to library quality control. (B) Percentages of nucleotide positions that were observed in the fitness profile and percentages of total single nucleotide mutations that were observed in the fitness profile after library quality control (mutation frequency of >0.1% in the DNA library). (C) Percentages of one, two, or all three nucleotide mutations observed for all of the positions prior to library quality control. (D) Percentages of one, two, or all three nucleotide mutations observed for all of the positions after library quality control. Download Figure S2, TIF file, 22.9 MB

Figure S3  Growth capacity of wild-type and ΔPB1-F2 mutant viruses. The TCID_50_s of the wild-type and PB1-F2 knockout (ΔPB1-F2) viruses are shown in log_10_. No significant difference in viral growth was detected. The ΔPB1-F2 mutant virus was generated by mutating the start codon of the gene (T120C) and introducing two stop codons at positions 12 (C153G) and 58 (G291A) (41, 42). Download Figure S3, TIF file, 22.9 MB

Figure S4  RF indexes of silent and nonsense mutations. The RF indexes of silent and nonsense mutations are shown in a box plot. The average RF index of silent mutations is significantly greater than that of nonsense mutations (two-tailed *t* test, *P* = 4.6E-021). Download Figure S4, TIF file, 22.9 MB

Figure S5  Protein stability predictions by I-Mutant and Rosetta. (A) Proportions of mutations that are destabilizing, neutral, or stabilizing in the PB1 protein as predicted by I-mutant 2.0 and shown as a pie chart. (B) Distribution of the SASAs of destabilizing and other mutations. The destabilizing mutations had significantly smaller SASAs (two-tailed *t* test, *P* = 5.4E-117). (C) Correlation of ΔΔG prediction results of I-Mutant and Rosetta. The absolute correlation coefficient of the predictions of these two methods is 0.3. Note that the sign of ΔΔG is opposite in these two computational tools. Download Figure S5, TIF file, 23.7 MB

Figure S6  Pairwise structural alignment of the PB1 protein and interface prediction. (A) Pairwise alignment of the PB1 protein and HCV NS5B (PDB code 2XI3). PB1 is gray, and NS5B is crimson. (B) Possible interfaces between PB1 and PA or PB2 are shown. The PA structure is shaded in purple, and the possible interacting residues of PB1 are red. The PB2 structure is shaded in green, and the possible interacting residues of PB1 are blue. The interface residues were predicted by the SASA changing upon complex formation with Sppider (84). Download Figure S6, TIF file, 23.7 MB

Figure S7  Nuclear localization of the PB1 and PA proteins upon PB1 mutations. (A and C) Quantification of the cellular localization of the PB1 (A) and PA (C) proteins of the wild-type and mutant viruses. A549 cells were infected with the wild-type or mutated virus at an MOI of 0.5. Cells were fixed and subjected to IFA at 8 h postinfection. At least 50 cells were analyzed for each mutant. Images were processed by ImageJ. (B) Example images of the cellular localization of PA proteins of PB1 mutant viruses. Download Figure S7, TIF file, 23.6 MB

Table S1  All of the vRdRp structures selected for homologous-structure analysis. The 20 vRdRp structures that we collected for homologous-structure analysis are presented. Four of them were filtered from multiple structure analysis because of a TM-score of <0.5 when aligned individually with the PB1 protein sequence. Table S1, DOCX file, 0.03 MB

Table S2  Phylogenetic analysis of amino acid positions 193, 197, and 198. The natural occurrence and *dN*/*dS* ratios of amino acids D193, K197, and K198 are shown. Table S2, DOCX file, 0.01 MB

Data Set S1  (A) PB1 fitness profiling results. The RF index of each PB1 point mutation in this study is shown. (B) Prediction of PB1 protein stability after single amino acid substitutions. Shown are the stability prediction data obtained by I-Mutant 2.0 for all of the single amino acid substitutions profiled and those obtained by Rosetta for all of the deleterious mutations. (C) Protein interface prediction results. Interfaces between PB1 and PA and between PB1 and PB2 were predicted with Sppider (residues with at least a 4% decrease in SASA and a >5-Å^2^ exposed surface area upon complex formation). Download Data Set S1, XLS file, 0.8 MB
