# Supplementary figures and images for: Annotating Protein Functional Residues by Coupling High-Throughput Fitness Profile and Homologous-Structure Analysis
Source: mBio. 2016 Nov 1;7(6):e01801-16. doi: 10.1128/mBio.01801-16 (PMC5090041; doi:10.1128/mBio.01801-16)

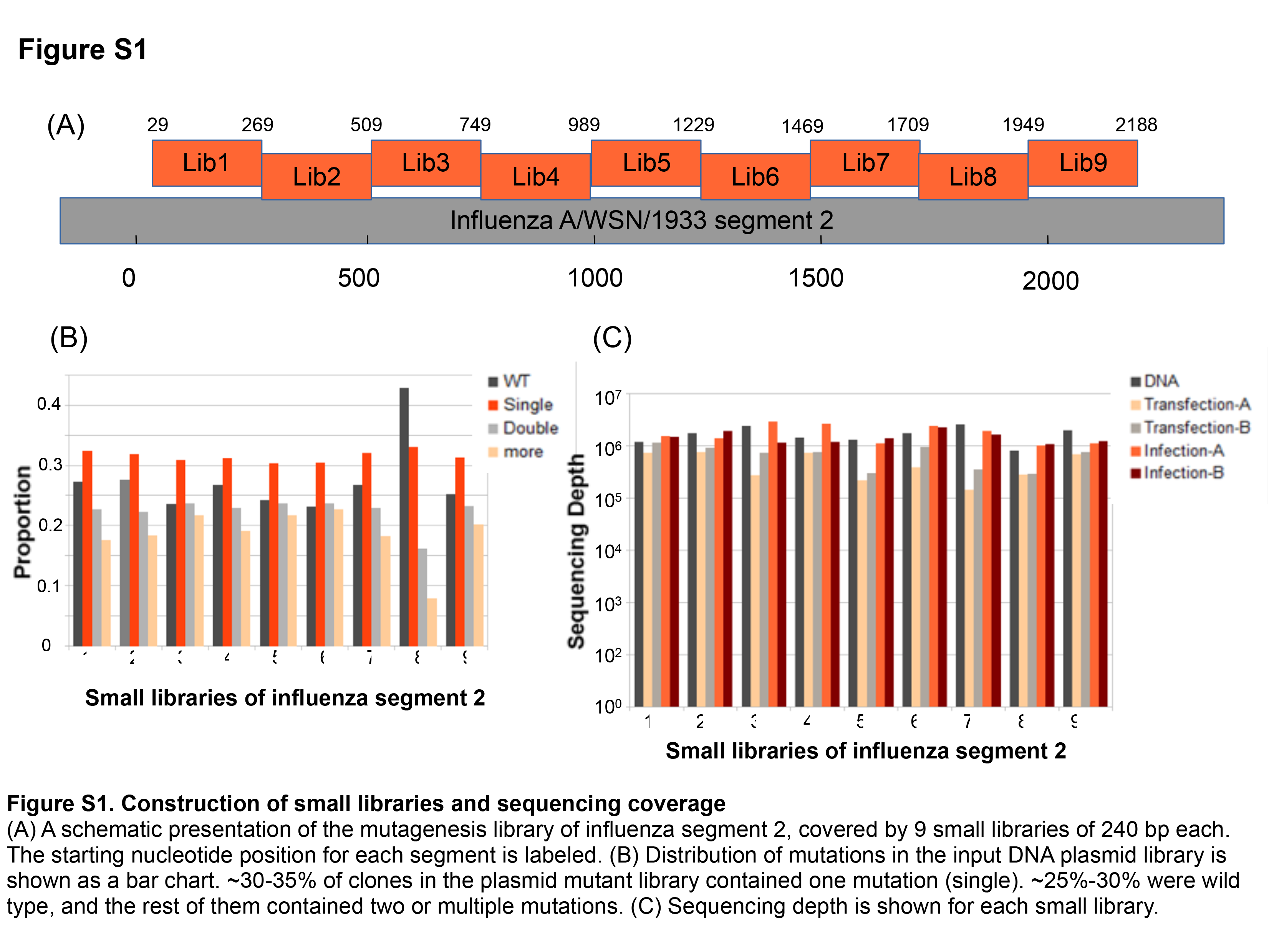

Supplement: Figure S1 — Construction of small libraries and sequencing coverage. (A) Schematic presentation of the mutagenesis library of influenza virus segment 2, covered by nine small libraries of 240 bp each. The starting nucleotide position of each segment is labeled. (B) Distribution of mutations in the input DNA plasmid library shown as a bar chart. From ~30 to 35% of the clones in the plasmid mutant library contained one mutation (single). From ~25 to 30% were wild type, and the rest of them contained two or more mutations. (C) Sequencing depth of each small library. Download [file mbo005163051sf1.tif]

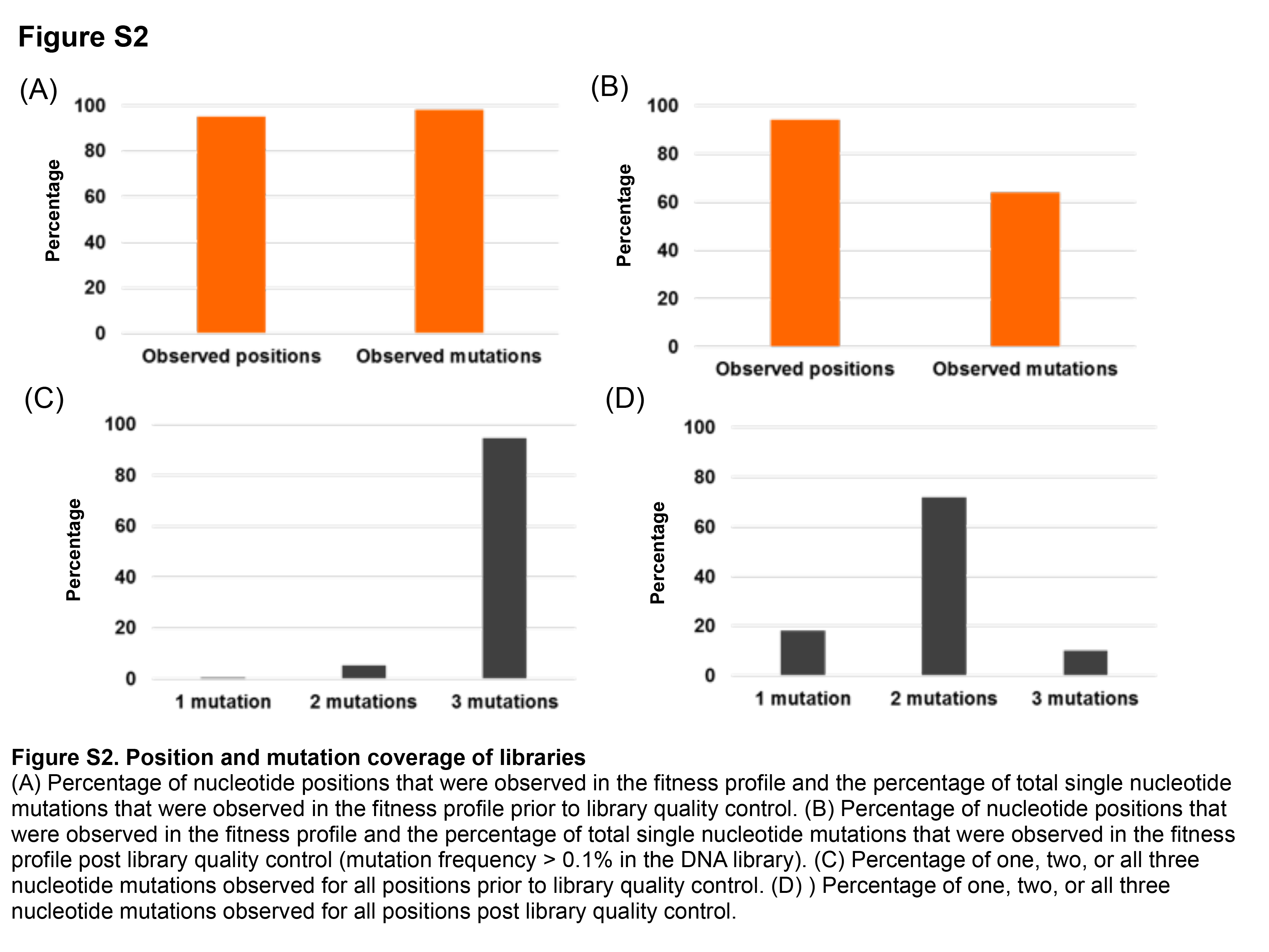

Supplement: Figure S2 — Positions and mutation coverages of libraries. (A) Percentages of nucleotide positions that were observed in the fitness profile and percentages of total single nucleotide mutations that were observed in the fitness profile prior to library quality control. (B) Percentages of nucleotide positions that were observed in the fitness profile and percentages of total single nucleotide mutations that were observed in the fitness profile after library quality control (mutation frequency of >0.1% in the DNA library). (C) Percentages of one, two, or all three nucleotide mutations observed for all of the positions prior to library quality control. (D) Percentages of one, two, or all three nucleotide mutations observed for all of the positions after library quality control. Download [file mbo005163051sf2.tif]

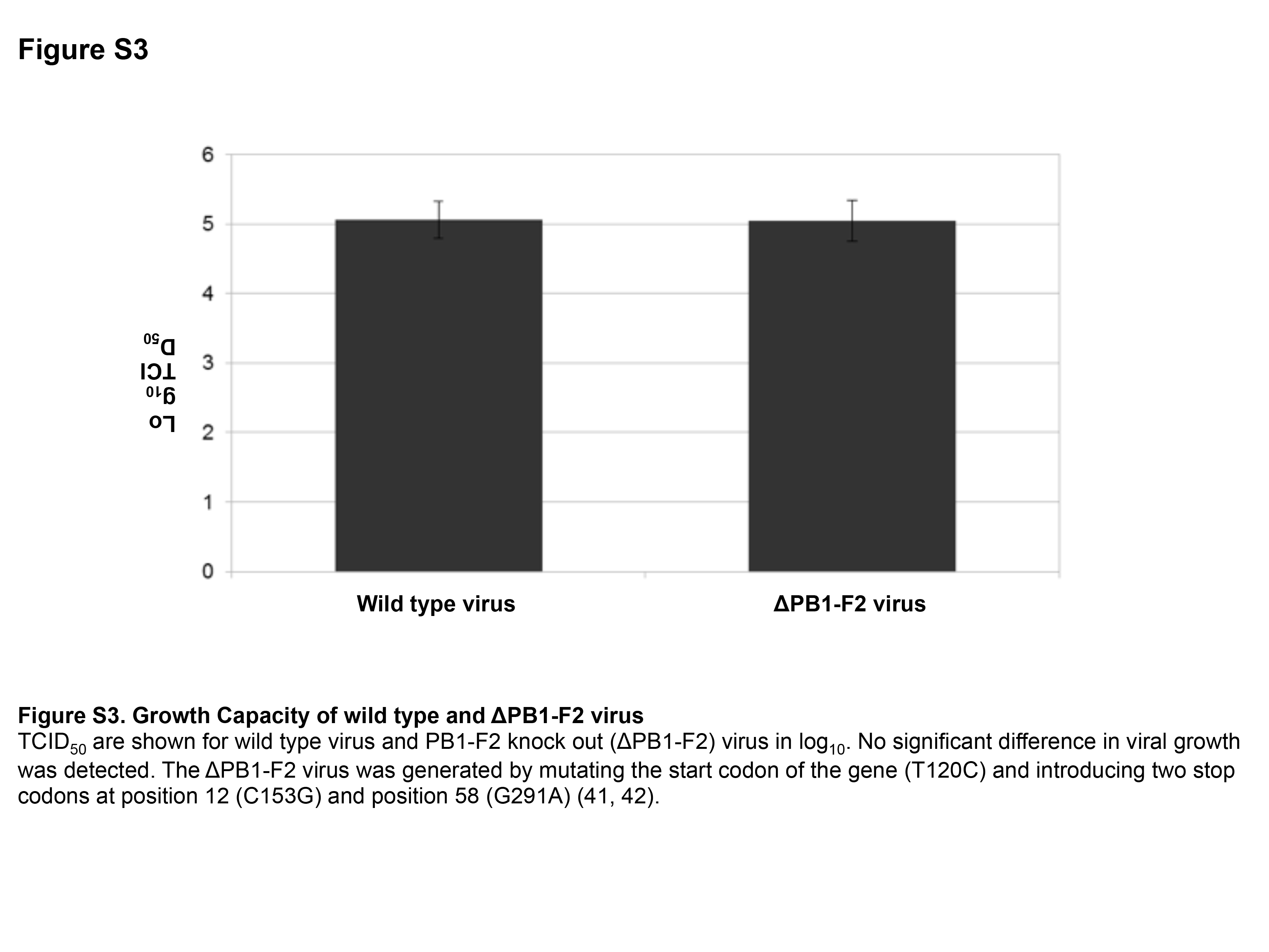

Supplement: Figure S3 — Growth capacity of wild-type and ΔPB1-F2 mutant viruses. The TCID50s of the wild-type and PB1-F2 knockout (ΔPB1-F2) viruses are shown in log10. No significant difference in viral growth was detected. The ΔPB1-F2 mutant virus was generated by mutating the start codon of the gene (T120C) and introducing two stop codons at positions 12 (C153G) and 58 (G291A) (41, 42). Download [file mbo005163051sf3.tif]

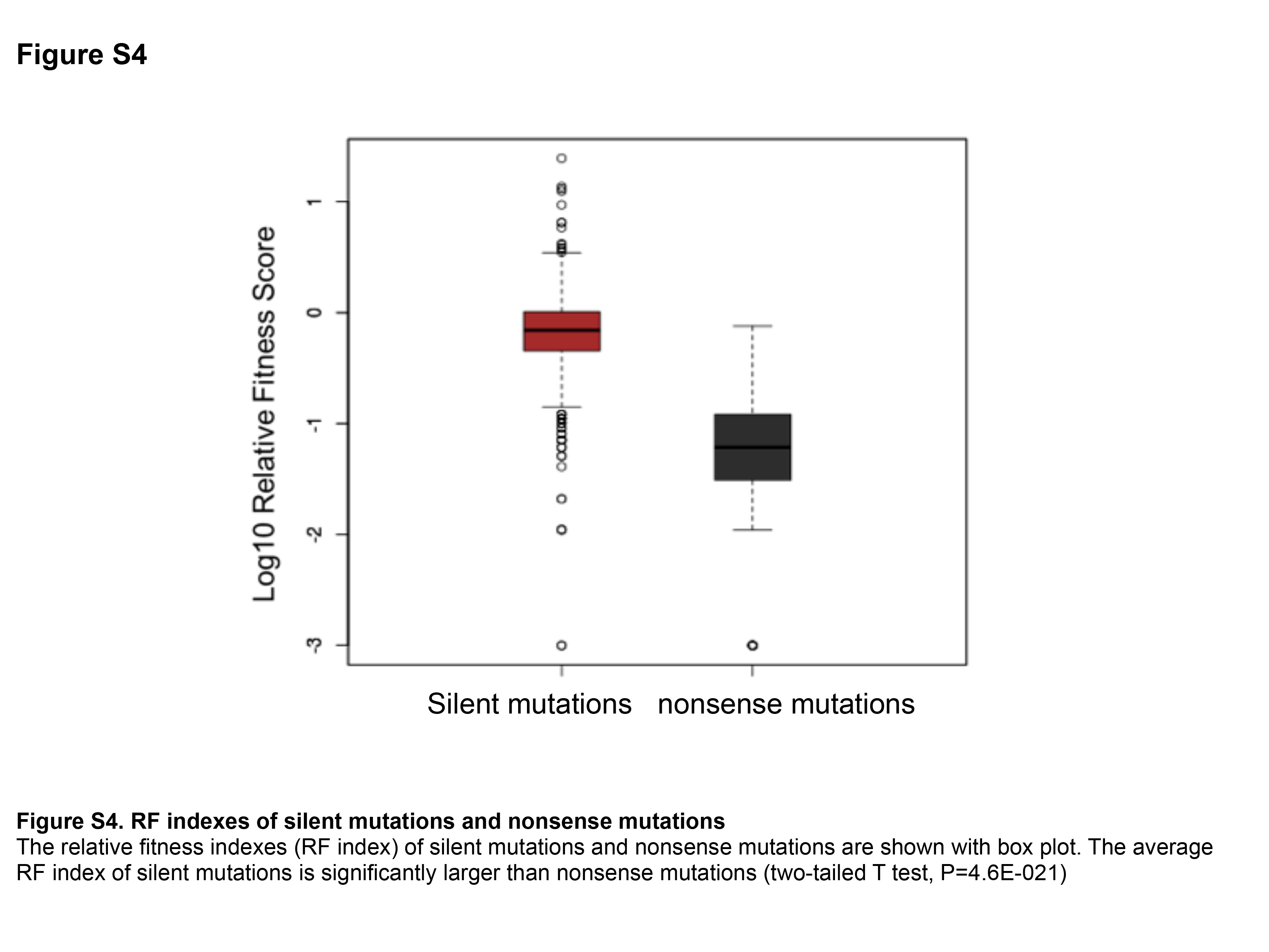

Supplement: Figure S4 — RF indexes of silent and nonsense mutations. The RF indexes of silent and nonsense mutations are shown in a box plot. The average RF index of silent mutations is significantly greater than that of nonsense mutations (two-tailed t test, P = 4.6E-021). Download [file mbo005163051sf4.tif]

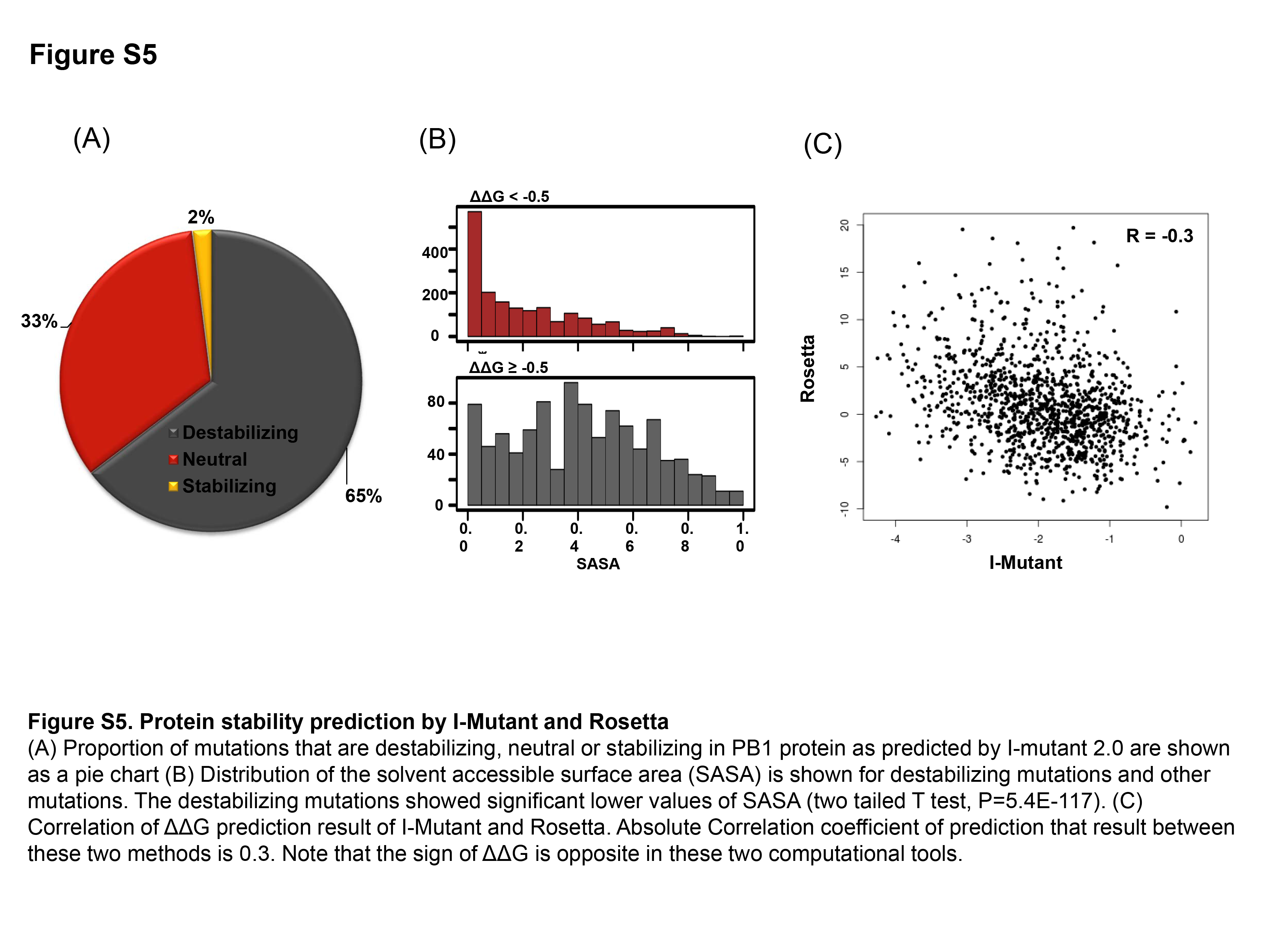

Supplement: Figure S5 — Protein stability predictions by I-Mutant and Rosetta. (A) Proportions of mutations that are destabilizing, neutral, or stabilizing in the PB1 protein as predicted by I-mutant 2.0 and shown as a pie chart. (B) Distribution of the SASAs of destabilizing and other mutations. The destabilizing mutations had significantly smaller SASAs (two-tailed t test, P = 5.4E-117). (C) Correlation of ΔΔG prediction results of I-Mutant and Rosetta. The absolute correlation coefficient of the predictions of these two methods is 0.3. Note that the sign of ΔΔG is opposite in these two computational tools. Download [file mbo005163051sf5.tif]

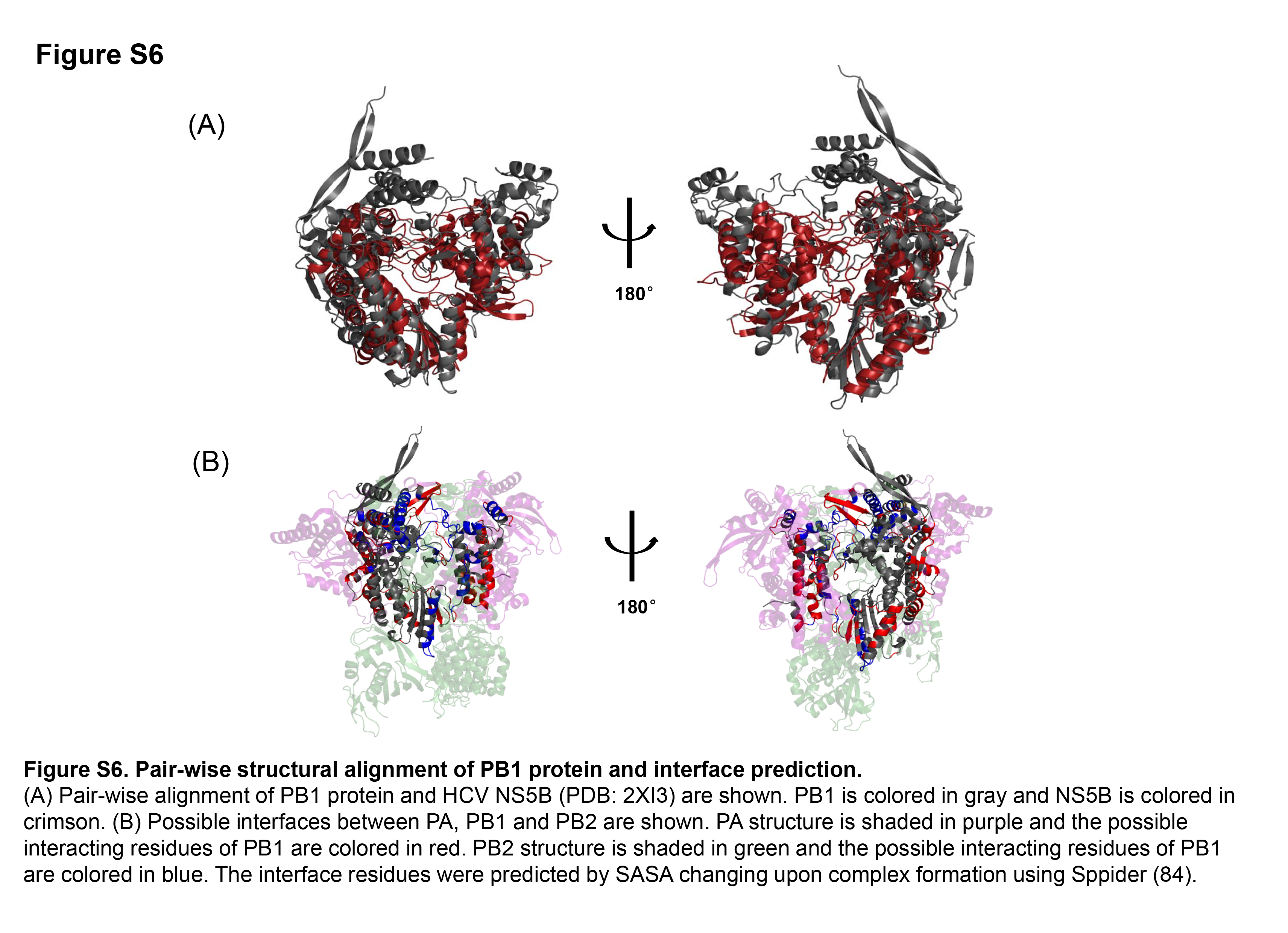

Supplement: Figure S6 — Pairwise structural alignment of the PB1 protein and interface prediction. (A) Pairwise alignment of the PB1 protein and HCV NS5B (PDB code 2XI3). PB1 is gray, and NS5B is crimson. (B) Possible interfaces between PB1 and PA or PB2 are shown. The PA structure is shaded in purple, and the possible interacting residues of PB1 are red. The PB2 structure is shaded in green, and the possible interacting residues of PB1 are blue. The interface residues were predicted by the SASA changing upon complex formation with Sppider (84). Download [file mbo005163051sf6.tif]

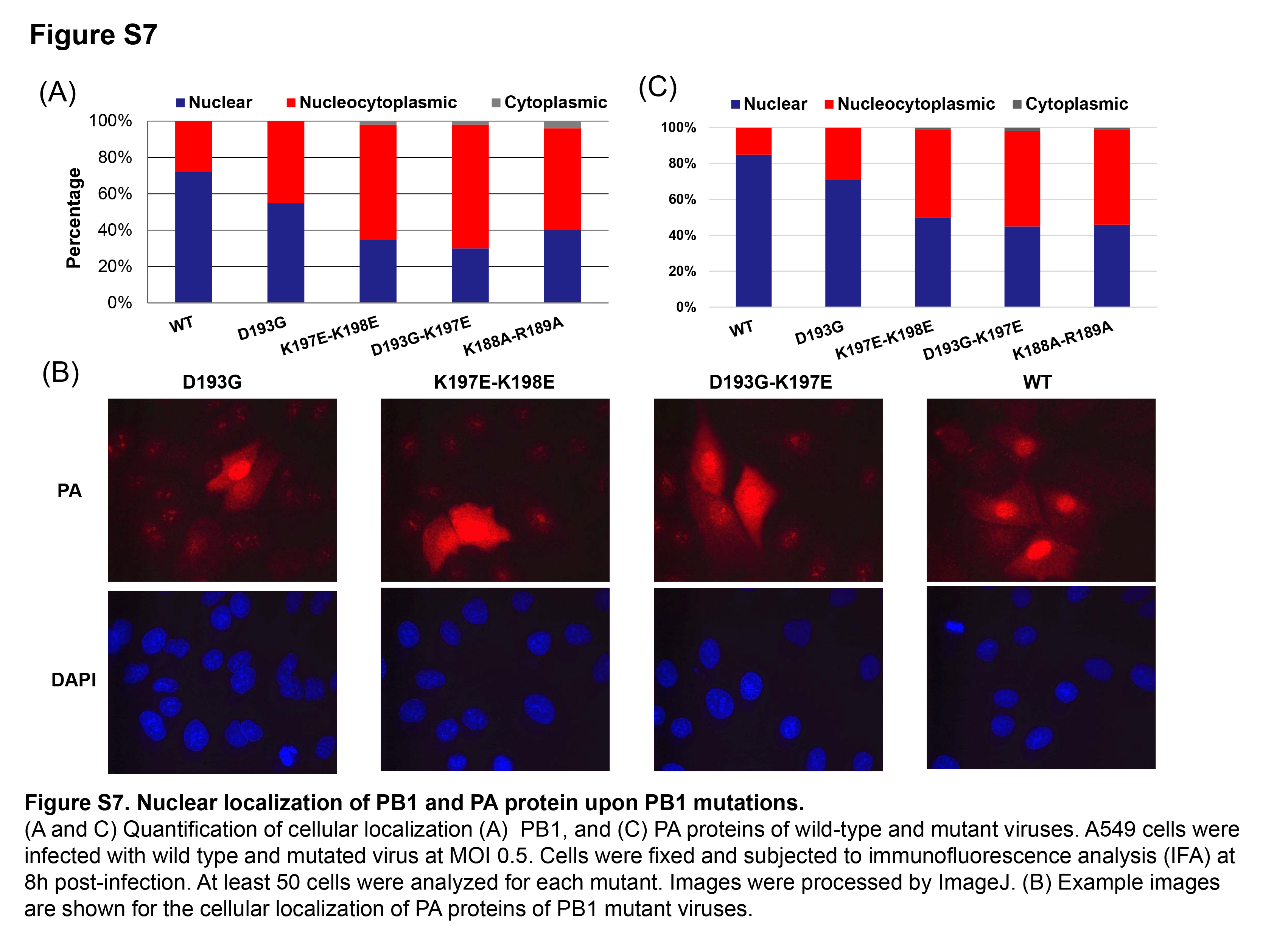

Supplement: Figure S7 — Nuclear localization of the PB1 and PA proteins upon PB1 mutations. (A and C) Quantification of the cellular localization of the PB1 (A) and PA (C) proteins of the wild-type and mutant viruses. A549 cells were infected with the wild-type or mutated virus at an MOI of 0.5. Cells were fixed and subjected to IFA at 8 h postinfection. At least 50 cells were analyzed for each mutant. Images were processed by ImageJ. (B) Example images of the cellular localization of PA proteins of PB1 mutant viruses. Download [file mbo005163051sf7.tif]
