# Supplementary material for: Annotating Protein Functional Residues by Coupling High-Throughput Fitness Profile and Homologous-Structure Analysis
Source: mBio. 2016 Nov 1;7(6):e01801-16. doi: 10.1128/mBio.01801-16 (PMC5090041; doi:10.1128/mBio.01801-16)
Supplement: Table S1 — All of the vRdRp structures selected for homologous-structure analysis. The 20 vRdRp structures that we collected for homologous-structure analysis are presented. Four of them were filtered from multiple structure analysis because of a TM-score of <0.5 when aligned individually with the PB1 protein sequence. [file mbo005163051st1.docx]

| **Baltimore class** | **family** | **genus** | **virus** | **abbreviation** | **PDB** | **Include in MSA** | **Reference** |
| --- | --- | --- | --- | --- | --- | --- | --- |
| +ssRNA viruses | Caliciviriade | Lagovirus | Rabbit hemorrhagic disease virus | RHEV | 1KHV | Yes | (62) |
|  |  | Norovirus | Murine norovirus | MuNORV1 | 3UQS | Yes | (63) |
|  |  |  | Norovirus | NORV | 3BSO | Yes | (64) |
|  |  | Sapovirus | Sapporo virus | SappV | 2CKW | Yes | (65) |
|  | Flaviviridae | Flavivirus | Dengue virus 3 | DENV3 | 4HHJ | Yes | (66) |
|  |  |  | Japanese encephalitis virus | JEV | 4K6M | No | (77) |
|  |  | Hepacivirus | Hepatitis C virus 1 | HCV1 | 2XI3 | Yes | (67) |
|  |  | Pestivirus | Bovine viral diarrhea virus | BVDV1 | 2CJQ | Yes | (68) |
|  | Leviviridae | Allolevivirus | Enterobacterio phage Qβ | Qβ | 3AVX | No | (78) |
|  | Picornaviridae | Aphthovirus | Foot and mouth disease virus | FMDV | 1U09 | Yes | (81) |
|  |  | Enterovirus | Humane rhinovirus 16 A | HuRV16A | 1XR7 | Yes | (70) |
|  |  |  | Coxsackie virus B3 | CoxVB3 | 3CDW | Yes | (71) |
|  |  |  | Humane rhinovirus 1B | HuRV1B | 1XR6 | Yes | (70) |
|  |  |  | Poliovirus 1 | PolV | 3OLB | Yes | (72) |
| ds RNA viruses | Birnaviridae | Aquabirnavirus | Infectious pancreatic necrosis virus | IPNV | 2YI9 | No | (79) |
|  |  | Avibirnavirus | Infectious bursal disease virus | IBDV | 2PUS | No | (80) |
|  | Cystoviridae | Cystovirus | Pseudomonas phage phi6 | Φ6 | 4A8O | Yes | (73) |
|  | Reoviridae | Orthoreovirus | Mammalian orthoreovirus 3 | MORV3 | 1N35 | Yes | (74) |
|  |  | Rotavirus | Simian rotavirus Sa11 | SRV | 2R7W | Yes | (75) |
| -ss virus | Bunyaviridae | Bunyavirus | La Crosse Bunyavirus | LACV | 5AMQ | Yes | (76) |

## Table S1. All vRdRp structure selected for homologous structure analysis

A list of 20 vRdRp structures that we collected for homologous structure analysis was presented. Four of them were filtered from multiple structure analysis (MSA) due to a TM-score < 0.5 when aligned individually with PB1 protein.
