# Supplementary material for: Annotating Protein Functional Residues by Coupling High-Throughput Fitness Profile and Homologous-Structure Analysis
Source: mBio. 2016 Nov 1;7(6):e01801-16. doi: 10.1128/mBio.01801-16 (PMC5090041; doi:10.1128/mBio.01801-16)
Supplement: Table S2 — Phylogenetic analysis of amino acid positions 193, 197, and 198. The natural occurrence and dN/dS ratios of amino acids D193, K197, and K198 are shown. [file mbo005163051st2.docx]

| **Position** | **Amino Acid** | **Occurrence Frequency** | **dN/dS** |
| --- | --- | --- | --- |
| 193 | D | 1 | 0.015265 |
| 197 | K | 0.992675 | 0.184951 |
| 198 | K | 0.995223 | 0.110705 |

## Table S2. Phylogenetic analysis of amino acid position 193, 197 and 198.

The natural occurrence and dN/dS of amino acid D193, K197 and K198 were listed.
